# Digital Health Professions Education on Diabetes Management: Systematic Review by the Digital Health Education Collaboration

**DOI:** 10.2196/12997

**Published:** 2019-02-21

**Authors:** Zhilian Huang, Monika Semwal, Shuen Yee Lee, Mervin Tee, William Ong, Woan Shin Tan, Ram Bajpai, Lorainne Tudor Car

**Affiliations:** 1 Centre for Population Health Sciences (CePHaS) Lee Kong Chian School of Medicine Nanyang Technological University Singapore Singapore; 2 Institute for Health Technologies (HealthTech NTU) Interdisciplinary Graduate School Nanyang Technological University Singapore Singapore Singapore; 3 Lee Kong Chian School of Medicine Nanyang Technological University Singapore Singapore Singapore; 4 School of Mechanical Aerospace and Engineering Nanyang Technological University Singapore Singapore Singapore; 5 Health Services and Outcomes Research Department National Healthcare Group Singapore Singapore; 6 Family Medicine and Primary Care Lee Kong Chian School of Medicine Nanyang Technological University Singapore Singapore

**Keywords:** evidence-based practice, health personnel, learning, systematic review, diabetes mellitus

## Abstract

**Background:**

There is a shortage of health care professionals competent in diabetes management worldwide. Digital education is increasingly used in educating health professionals on diabetes. Digital diabetes self-management education for patients has been shown to improve patients’ knowledge and outcomes. However, the effectiveness of digital education on diabetes management for health care professionals is still unknown.

**Objective:**

The objective of this study was to assess the effectiveness and economic impact of digital education in improving health care professionals’ knowledge, skills, attitudes, satisfaction, and competencies. We also assessed its impact on patient outcomes and health care professionals’ behavior.

**Methods:**

We included randomized controlled trials evaluating the impact of digitalized diabetes management education for health care professionals pre- and postregistration. Publications from 1990 to 2017 were searched in MEDLINE, EMBASE, Cochrane Library, PsycINFO, CINAHL, ERIC, and Web of Science. Screening, data extraction and risk of bias assessment were conducted independently by 2 authors.

**Results:**

A total of 12 studies met the inclusion criteria. Studies were heterogeneous in terms of digital education modality, comparators, outcome measures, and intervention duration. Most studies comparing digital or blended education to traditional education reported significantly higher knowledge and skills scores in the intervention group. There was little or no between-group difference in patient outcomes or economic impact. Most studies were judged at a high or unclear risk of bias.

**Conclusions:**

Digital education seems to be more effective than traditional education in improving diabetes management–related knowledge and skills. The paucity and low quality of the available evidence call for urgent and well-designed studies focusing on important outcomes such as health care professionals’ behavior, patient outcomes, and cost-effectiveness as well as its impact in diverse settings, including developing countries.

## Introduction

Diabetes is one of the biggest global public health concerns affecting an estimated 425 million adults worldwide, and this number is expected to rise to 629 million by 2045 [1]. This is coupled with a shortage of health care professionals competent in delivering high-quality diabetes care [2,3]. Enhancing both the size and competencies of health care professionals is a priority and improving health professions education is seen as one of the key strategies to this end [4]. Digital education, broadly defined as the use of digital technology in education, has been recognized as having the potential to improve health professions education by making it scalable, interactive, personalized, global, and cost-effective [5-7].

Past systematic reviews on digital education have focused mainly on diabetes self-management education for patients, showing an improvement in patients’ knowledge and outcomes [8-10]. The effectiveness of digital education interventions for health care professionals on diabetes management is still unknown [11]. To address this gap, we performed a systematic review to evaluate the effect of digital education on diabetes management on health care professionals’ knowledge, skills, attitudes, competencies, and behaviors, as well as its impact on patient outcomes.

## Methods

### Systematic Review Guidance

We followed the Cochrane Handbook of Systematic Reviews for our methodology [12] and the Preferred Reporting Items for Systematic Reviews and Meta-Analyses statement for reporting [13]. For a detailed description of the methodology, please refer to the study by Car et al [14].

### Data Sources and Searches

This review is part of an evidence-synthesis initiative on digital health professions education, where an extensive search strategy was developed for a series of systematic reviews on different modalities of digital health education for health care professionals (see Multimedia Appendix 1) [15]. The following databases were searched from January 1990 to August 2017:

1. The Cochrane Central Register of Controlled Trials (The Cochrane Library,)
2. MEDLINE (Ovid)
3. EMBASE (Elsevier)
4. PsycINFO (Ovid)
5. Educational Resource Information Centre (ERIC; Ovid)
6. Cumulative Index to Nursing and Allied Health Literature (CINAHL; EBSCO)
7. Web of Science Core Collection (Clarivate analytics).

We included studies in all languages and at all stages of publication. Our search strategy included gray literature sources such as Google scholar, trial registries, theses, dissertations, and academic reports. The citations retrieved from different sources were combined into a single library and screened by 2 authors independently. We also screened references of included papers for potentially eligible studies. Discrepancies and disagreements were resolved through discussion until a consensus was reached.

### Study Selection

We included randomized controlled trials (RCTs), cluster RCTs, and quasi-RCTs and excluded cross-over trials due to high likelihood of a carry-over effect in this type of studies [12]. Studies on pre- or postregistration health care professionals taking part in digital education interventions on diabetes management were considered eligible. We defined health care professionals in line with the Health Field of Education and Training (091) in the International Standard Classiﬁcation of Education [16]. Studies on digital education on both type 1 and type 2 diabetes at all educational levels were included.

We defined digital education as any teaching and learning that occurs by means of digital technologies. We considered eligible all digital education modalities, including offline and online education, Serious Gaming and Gamification, Massive Open Online Courses, Virtual Reality Environments, Virtual Patient Simulations, Psychomotor Skills Trainers, and mobile learning. Eligible comparisons were traditional, blended, or another form of digital education intervention on diabetes management. Traditional education was defined as any teaching and learning taking place via nondigital educational material (eg, textbooks) or in-person human interaction (eg, lecture or seminar). Traditional education also included usual learning, for example, usual revisions as well as on-the-job learning without a specific intervention in postregistration health care professionals. Blended education was defined as the act of teaching and learning that combines aspects of traditional and digital education. Eligible primary outcomes measured using any validated and non-validated instruments were knowledge, skills, competencies, attitudes, and satisfaction. Eligible attitudes-related outcomes comprised all attitudes toward patients, new clinical knowledge, skills, and changes to clinical practice.

Eligible secondary outcomes included patient outcomes in studies on postregistration health care professionals (eg, patients’ blood pressure, blood glucose, and blood lipid levels), change in health care professional’s behavior (ie, treatment intensification, defined as an intensity or dose increase of an existing treatment or the addition of a new treatment/class of medication), and economic impact of the intervention.

### Data Extraction

In this study, 2 authors independently extracted data from studies using a structured and piloted data extraction form. We extracted information on study design, participants’ demographics, type, content and delivery of digital education, and information pertinent to the intervention. Study authors were contacted in case of unclear or missing information.

### Risk of Bias and Quality of Evidence Assessment

The methodological quality of included RCTs was independently assessed by 2 authors using the Cochrane Risk of Bias Tool [12]. The risk of bias assessment was piloted between the reviewers, and we contacted study authors in case of any unclear or missing information. We assessed the risk of bias in included RCTs for the following domains: (1) random sequence generation; (2) allocation concealment; (3) blinding of participants to the intervention; (4) blinding of outcome assessment; (5) attrition; (6) selective reporting; and (7) other sources of bias [17]. Cluster RCTs were assessed using 5 additional domains: (1) recruitment bias; (2) baseline imbalance; (3) loss of clusters; (4) incorrect analysis; and (5) comparability with individually randomized trials [12].

### Data Synthesis and Analysis

In line with Miller’s classification, a learning model for assessment of clinical competence [18], we classified outcomes based on the type of outcome measurement instruments used in the study. For example, multiple-choice questionnaires were classified as assessing knowledge and objective structured clinical examinations as assessing participants’ skills.

Although some studies reported change scores, we presented only postintervention data as those were more commonly reported and to ensure consistency and comparability of findings. Continuous outcomes are presented using mean difference (for outcomes measured using the same measurement tool), standardized mean difference (SMD; for outcomes measured using diverse measurement tools), and 95% CIs. Dichotomous outcomes are presented using risk ratios (RRs) and 95% CIs. As we were unable to identify a clinically meaningful interpretation of effect size in the literature for digital education interventions, we interpreted the effect size using Cohen *rule of thumb* with SMD greater than or equal to 0.2 representing a small effect, SMD greater than or equal to 0.5 a moderate effect, and SMD greater than or equal to 0.8 a large effect [19,20]. In studies that reported more than one measure for each outcome, the primary measure, as defined by the primary study authors, was considered.

### Heterogeneity and Subgroup Analyses

Heterogeneity was assessed qualitatively using information relating to participants, interventions, controls, and outcomes as well as statistically using the I^2^ statistic for outcomes allowing for pooled analysis [17]. Due to substantial methodological, clinical, and statistical heterogeneity (I^2^>50%), we conducted a narrative synthesis according to type of comparison, that is, (1) digital education versus traditional education, (2) digital education versus blended education, and (3) one digital education type versus another digital education type. Subgroup analyses were not feasible owing to the small number of studies and limited information. We presented the study findings in a forest plot using the random effects model and standardized mean difference as the measurement scales were different and without the pooled estimates.

## Results

### Included Studies

**Figure 1:**
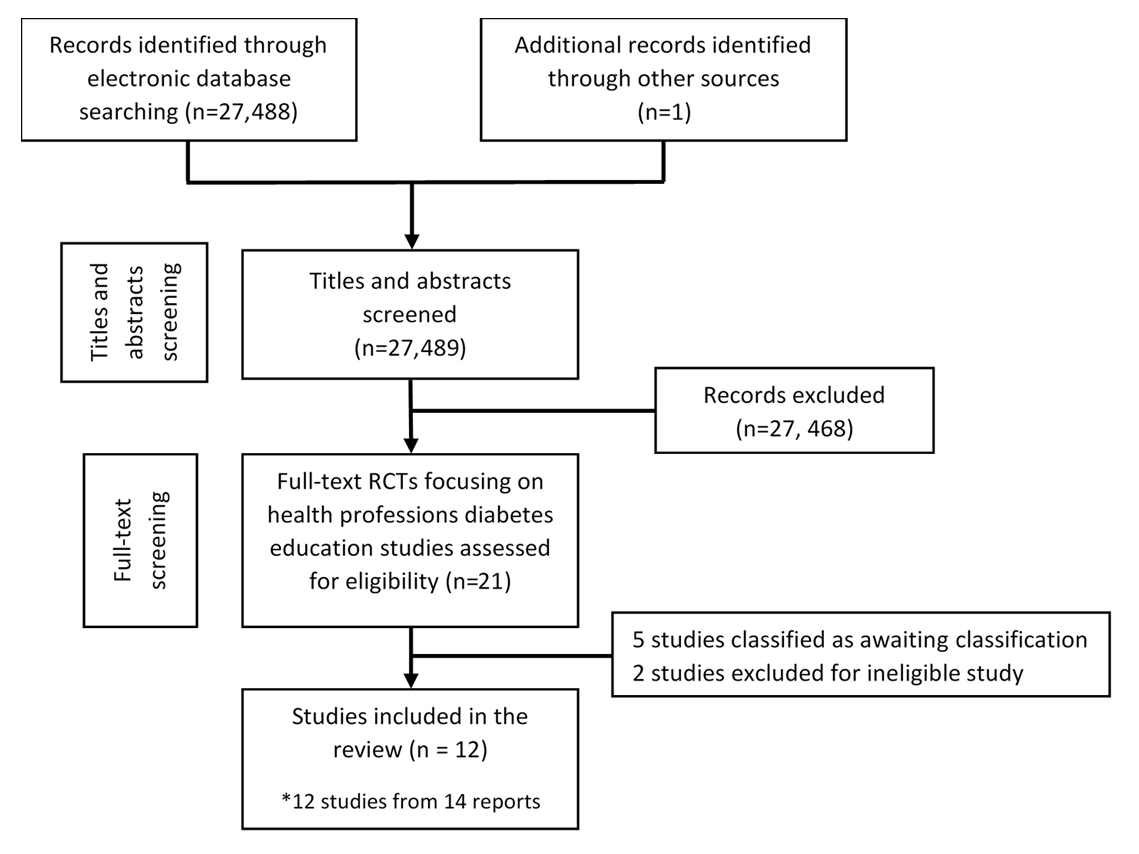
Preferred Reporting Items for Systematic Reviews and Meta-Analyses flowchart of included studies.

Our search strategy for a series of systematic reviews focusing on different digital health professions education modalities yielded 30,532 unique references. We removed 459 duplicates, and upon screening of titles and abstracts, the screening excluded 30,050 citations. We identified 23 potentially eligible studies for which we retrieved and screened full texts. Of these, we included 12 studies: 9 RCTs and 3 cluster RCTs, all published in English (Figure 1). Moreover, 1 study was reported by 3 journal papers [21-23]. Although presented as a cluster RCT, this study included randomization at the individual, physician level and was therefore considered an RCT. A total of 9 studies were excluded due to ineligible study design (n=3), missing data (n=5), and ineligible participants (n=1; Figure 1).

### Participant Characteristics

There were 2263 health care professionals in 12 included studies [23-34]. A third of the studies included less than 50 participants. The study with 3 published reports had 1182 patient records as a measure of clinical outcomes [21-23]. Only 1 study targeted pediatric patients with type 1 diabetes [28]. All other studies reporting patient outcomes focused on adult patients with type 2 diabetes. A total of 8 studies focused on doctors [23,24,26,27,29,32-34]. Moreover, 1 study each focused on medical students [30], pharmacy students [25], nurses [31], and jointly on doctors, nurses, and dietitians [28].

### Study Characteristics

A total of 10 studies were conducted in high-income countries including Australia [30], the United States [23,24,26,29,31-33], and the United Kingdom [27,28]. A total of 2 studies were conducted in middle-income countries such as Thailand [25] and Brazil [34] each.

A total of 6 studies compared digital education with traditional education [25,26,29,30,32,34]. A total of 3 studies compared 2 different methods of digital education interventions [23,27,31], 2 compared blended education with usual education [28,33], and 1 study with 3 arms compared usual, blended, and digital education [24]. Only 4 studies reported duration of the intervention lasting from an hour to 2 weeks [25,26,30,34].

Various types of modalities were used to deliver the digital education interventions. A total of 3 studies used a Web-based or online portal [23,27,28]; 3 used a scenario-based simulation software [24,26,32]; 1 study each assessed high-fidelity mannequins [31]; an online game app on the computer [34]; periodic email reminders on the lecture content [33]; personal digital assistant–delivered learning materials [29]; and a computer-based diabetes management program [25].

All studies except 3 employed clinical scenarios in the digital education intervention [21,24-27,30-32,34]. The remaining 3 studies used text-based learning [29,33,28]. Feedback was provided to participants in the intervention group in 7 studies [21,24,26-28,32,34]. A total of 2 studies comparing different forms of digital education reminded participants to log into the system [21,27], whereas one employed an email reminder to consolidate learned knowledge [33]. Half of the studies evaluated interactive digital education interventions [21,24,26,31,32,34].

Comparison interventions were also varied; 3 studies utilized a Web-based system (online portal) for the control group [23,27,31]; 4 compared the digital education intervention with face-to-face education [25,29,33,34]; 1 provided hard copy materials [29]; 1 reported *revision as usual* where participants could access relevant materials available to them [30]. A total of 4 studies focusing on postregistration education did not include any control intervention [24,26,28,32].

A total of 11 studies measured primary outcomes; 6 assessed knowledge with questionnaires [25,27,29,31-33]; 5 assessed skills and competency (measured as a combination of knowledge and skill) [25,28,30,32,34]; 2 assessed learners’ attitude [27,34]; and 4 assessed learners’ satisfaction [24,26,30,34]. A total of 5 studies measured secondary outcomes; 2 assessed the cost of the intervention [26,28]; 4 assessed patient outcomes (ie, patients meeting glycated hemoglobin [23,24,26,28]; low-density lipoprotein [23,24,26]; and blood pressure control [23,26] goals); and 2 assessed treatment intensification (intensifying the treatment regimen as required) [21,24].

Participant type and content of diabetes education across the studies varied widely and included diabetes management skills for primary care physicians (PCPs) [23,24,26,27,34]; diabetes clinical care for primary care, family, and internal medicine residents [29,32]; communication skills for pediatric doctors, nurses, and dieticians managing type 1 diabetes patients [28]; clinical endocrinology skills for medical students [30]; primary care residents’ training on Hepatitis B vaccination for diabetes patients [33]; nursing care for hypoglycemic patients [31]; and diabetes management knowledge, communication, and patient note writing skills for pharmacy students [25].

### Risk of Bias in the Included Studies

**Figure 2:**
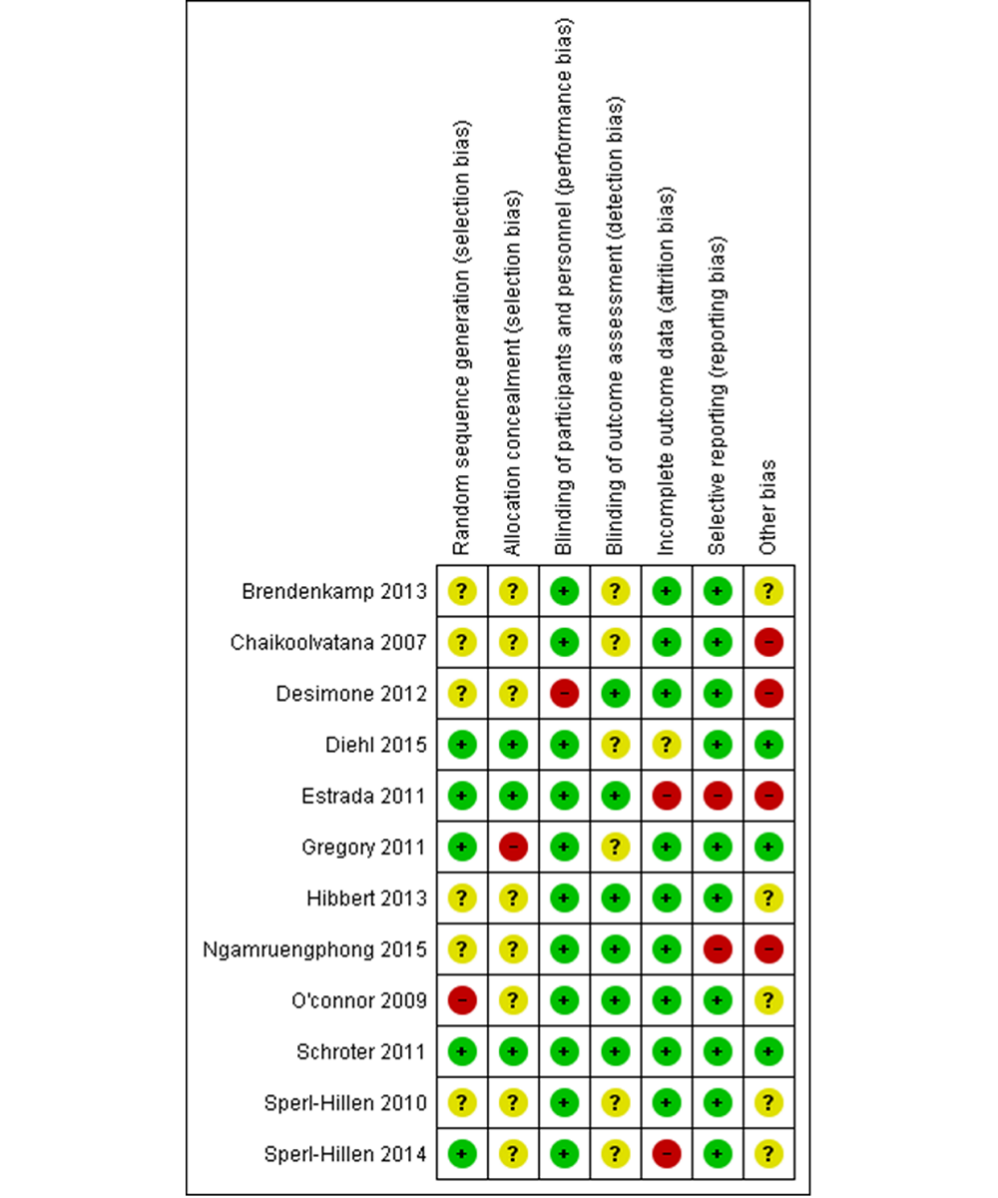
Risk of bias summary: review authors' judgement about each risk of bias item for each included study. The symbol "+" indicates a low risk of bias, "?" indicates unclear risk of bias and "-" indicates a high risk of bias. The methodology of 2 studies (Crenshaw 2010 and Billue 2012) are duplicated with (Estrada 2011) and not presented in this figure.

Of 12 included studies, 7 were judged at a high risk of bias and three studies had an unclear risk of bias for at least three domains. Of three cluster RCTs, two were judged at a high risk due to baseline imbalance (Figure 2, Multimedia Appendix 2).

### Digital Education Versus Traditional Education

A total of 4 RCTs [25,29,30,34] and 2 cluster RCTs [26,32] compared digital education with traditional education, including no intervention (ie, knowledge acquisition as usual or usual on-the-job training), face-to-face lectures or hard copy printouts (Table 1). A total of 3 studies measured knowledge outcome. Of these studies, 2 compared online virtual simulation and computer-based learning intervention with no intervention, respectively, and reported moderate-to-large postintervention knowledge gain in the digital education group compared with the control group (Multimedia Appendix 3 and Multimedia Appendix 4). The final study compared learning materials either printed or displayed on a mobile electronic device for medical residents, where no between-group difference in postintervention knowledge scores was found [29].

Skills were assessed in 4 studies [25,30,32,34], which largely reported higher effectiveness of digital education interventions (Multimedia Appendix 3 and Multimedia Appendix 4). Moreover, 1 study comparing a training video and no intervention (usual revision) for medical students reported significant improvement in lower limb examination (RR: 2.29; 95% CI 1.05-4.99) and diabetes history taking skills (RR: 4.17; 95% CI 1.18-14.77), and no difference in thyroid disease examination. Another study comparing computer-based and face-to-face learning for final year pharmacy students found large improvements in subjective, objective, assessment, and plan note writing skills (SMD 0.78; 95% CI 0.33-1.22) in the digital education group and no difference in patient history taking skills between the groups [25]. The third study, comparing an online virtual case-based simulation with no intervention for medical residents, reported higher proportion of patients meeting safe treatment goals in 3 out of 4 hypothetical simulation cases [32] (Multimedia Appendix 4). The final study compared an online game with face-to-face learning for PCPs and assessed their competency, that is, a combination of factual knowledge and problem-solving skills of PCPs on insulin therapy for diabetes reported small improvements in the digital education group (SMD: 0.4; 95% CI 0.09-0.70]) [34].

Only 1 study comparing a simulated physician learning software and no intervention measured patient clinical outcomes and cost [24]. The study reported the mean pre- and postintervention change (95% CI) in glycated hemoglobin, systolic blood pressure, diastolic blood pressure, and low-density lipoprotein levels of patients under the care of participating physicians. Improvements were observed for all measures from baseline to postintervention in both intervention and control groups. However, when comparing the groups, results were mixed (Multimedia Appendix 4) [24]. Cost savings of US $71 per patient was reported for the intervention group compared with the control group from the health plan perspective, but the difference was not statistically significant.

Learner’s satisfaction was assessed with self-reported surveys in 3 studies. Only 1 study focusing on online games evaluated satisfaction for both intervention and control groups, but the use of different questionnaires did not allow between-group comparisons [32]. The same study found significantly better diabetes management and insulin-related attitudes and beliefs toward the digital education intervention in the intervention group [32]. The remaining 2 studies assessed satisfaction only in the intervention group, and more than 80% of participants were satisfied with the digital intervention [24,28].

### Blended Education Versus Traditional Education

One cluster RCT [26] and one RCT [31] compared blended education with traditional education and evaluated knowledge, skills, patients’ glycated hemoglobin levels, and economic impact. The blended education within the RCT comprised the standard education and an additional 30-min didactic lecture, a pocket card, and monthly email reminders on lecture content. The study reported large improvement in postintervention knowledge score in the blended education group compared with the control group (SMD: 1.98; 95% CI 1.21-2.74) [31].

The blended learning program of the cluster RCT included Web-based training and practical workshops for behavioral change in pediatric patients with type 1 diabetes, whereas the control group received no intervention [26]. The blended education group had a large improvement in the postintervention communication skills score (SMD: 1.58; 95% CI 0.99-2.17) and a higher proportion of tasks done or partially done in shared agenda setting (RR: 7.49; 95% CI 1.88-29.9) compared with the control group (Multimedia Appendix 3). Cost differences in the mean total National Health Service cost (direct costs: training; indirect costs: clinic visits) were not statistically significant; although, the blended education intervention incurred an additional mean cost of £183.96 per patient. There was no statistically significant difference between the groups in patient outcomes (ie, glycated hemoglobin levels) and patients’ quality of life (Multimedia Appendix 4).

### Digital Education Versus Blended Education Versus Traditional Education

One RCT study compared digital education, blended education, and traditional education to improve the safety and quality of diabetes care delivered by PCPs [24]. The digital education group received online case-based simulation, and the blended education group also received feedback in the form of additional face-to-face physician opinion. Learners’ satisfaction and patient clinical outcomes (ie, mean change in glycated hemoglobin, blood pressure, and low-density lipoprotein level and treatment intensification) were assessed. Over 97% of PCPs who completed the education intervention rated their satisfaction with the digital education and blended interventions as excellent or very good after completing the simulated cases. The mean glycated hemoglobin level significantly improved in the digital education group compared with blended or traditional education (Multimedia Appendix 4). There was no statistically significant difference across the intervention groups in the remaining patient outcomes (Multimedia Appendix 4).

**Table 1:** Characteristics of the included studies.

Study, design, and country		Learning modality	Type of participants	Number of sites and participants	Intervention duration	Type of outcome
**Digital education versus traditional education**					
	Chaikoolvatana 2007 [25]; RCT^a^; Thailand	I^b^: Computer based learning (CBL); C^c^: face-to-face lectures	Final year pharmacy students	I: 43, C: 40	I: 2 hours; C: 2 3-hour sessions; (over 2 months)	(1) Knowledge; (2) skills
	Desimone 2012 [29]; RCT; United States	I: PDA^d^ version education materials; C: Printed materials	Internal medicine residents	I: 11, C: 11	Over 1 month	Knowledge
	Diehl 2017 [34]; RCT; Brazil	I: Online game; C: Face-to-face lectures and activities	Primary Care Physicians	I: 94, C: 76	4 hours (over 3 months)	Skills
	Hibbert 2013 [30]; RCT; Australia	I: Training Video; C: No intervention (usual revision)	Second year medical students	I: 12, C: 10	Over 2 Weeks	Skills
	Sperl-Hillen 2010 [26]; cRCT^e^; United States	I: Simulation Software; C: No intervention	Primary Care Physicians and their patients	I: 20 sites, (1847 patients), C: 21 sites, (1570 patients)	5.5 days; (over 6 months)	Patient outcomes; Economic impact
	Sperl-Hillen 2014 [32]; cRCT; United States	I: Simulation software; C: No intervention (Not assigned learning cases)	Family/ internal medicine residents	I: 10 sites (177 residents), C: 9 sites (164 residents)	Over 6 months	Knowledge; Skills
**Blended learning versus traditional education**					
	Gregory 2011 [28]; cRCT; United Kingdom	I: Web-based intervention and practical workshops; C: No intervention	Paediatric doctors, nurses, psychologists, dieticians, and their patients	I: 13 sites (356 patients), C: 13 sites (333 patients)	Over 12 months	Skills; Patient outcomes; Economic impact
	Ngamruengphong 2011 [33]; RCT; United States	I: Standard education+30 min didactic lecture, a pocket card, and monthly e-mail reminders that consisted of the lecture content; C: Standard residency education	Primary care residents	I: 20, C: 19	Over 2 months	Knowledge
**Digital education versus digital education**					
	Billue 2012 [21]; RCT United States; Estrada 2011[23]; RCT United States; Crenshaw 2010[22]; RCT; United States	I: Web-based intervention with feedback; C: Web-based intervention without feedback	Family/ general/ internal medicine physicians	I: 48 physicians (479 patients), C: 47 physicians (466 patients)	Over 2 years	Patient outcomes
	Brendenkamp 2013 [31]; RCT; United States	I: Simulation (High fidelity Mannequin); C: Web-based intervention	Staff nurses	I: 47, C: 49	Not reported	Knowledge
	Schroter 2011 [27]; RCT; United Kingdom	I: Web-based learning + Diabetes Needs assessment tool (DNAT); C: Web-based learning without DNAT	Diabetes doctors and nurses	I: 499, C: 498	Over 4 months	Knowledge
**Blended learning versus digital education versus traditional education**					
	O'Connor 2009 [24]; RCT; United States	Group A: No intervention; Group B: Simulated web-based learning; Group C: simulated case-based physician learning + physician opinion leader feedback	Primary care physicians and their patients	Group A: 100 physicians, 691 patients; Group B: 100 physicians, 725 patients; Group C: 99 physicians, 604 patients	Not reported	Patient outcomes

^a^RCT: randomized controlled trial.

^b^I: intervention group.

^c^C: control group.

^d^PDA: Personal Digital Assistance.

^e^cRCT: cluster RCT.

### Digital Education Versus Digital Education

A total of 3 RCT studies compared 2 different digital education modalities [21,25,29]. Moreover, 1 study compared a high-fidelity simulation mannequin with an online learning system [29]. The other 2 studies, employing the same Web-based (online) system in both the groups, evaluated the addition of an interactive learning needs assessment tool or feedback to the intervention group, respectively [21,25].

Studies reported no significant difference in terms of knowledge, attitudes, and patient outcomes. The study evaluating the use of feedback as part of the digital education intervention reported higher study engagement in the intervention group as reflected by the total number of pages viewed (SMD: 1.40; 95% CI 0.95-1.85), total number of visits (SMD: 1.38; 95% CI 0.93-1.83]), duration of Web access in min (SMD: 1.07; 95% CI 0.64-1.50), and the number of components viewed (SMD: 1.14; 95% CI 0.70-1.57) [18].

## Discussion

### Principal Findings

We found 12 studies evaluating the effectiveness of digital health professions education on diabetes management. Although evidence is limited, heterogeneous, and of low quality, our findings suggest that digital and blended education may improve health care professionals’ knowledge and skills compared with traditional education. However, an improvement in knowledge and skills does not seem to translate into improvements in diabetes care as reflected by little or no difference in sparsely reported patient outcomes in the included studies. Although simulated learning seems to be more effective in improving patient outcomes compared with the other strategies assessed, studies comparing different forms of digital education reported no statistically significant difference between groups.

The inconsistency between the effect on health care professionals’ and patients’ outcomes observed in our review is in line with the existing literature, where knowledge and skill gains outweigh improvements in patient outcomes [33]. Yet patient outcomes were only reported in 4 diverse studies in this review. The lack of patient-related data is common in digital education studies, possibly owing to difficulty in measuring patient outcomes, especially in preregistration health care professionals. Furthermore, patient outcomes are potentially affected by contextual factors unrelated to health care professionals’ competence, such as patients’ health beliefs and financial barriers [34]. Finally, a lack of difference between the groups observed in the included studies may be merely due to their insufficient statistical power to evaluate patient outcomes.

Although digital education has been present in health professions’ education for the last 2 decades, its technological development and adoption has been expedited in recent years [35], particularly in high-income countries. Likewise, most studies in our review were published since 2010 and are from high-income countries. Widespread access to digital technology in high-income countries may diminish the effects of digital education interventions in RCTs, given that blinding is not possible, and the control group participants may interact or have alternative electronic access to information. Studies on the use of digital education in low- and middle-income countries would provide a more comprehensive assessment as the technological setup and learning infrastructure is more limited [36-38]. Although there is a universal need for scalable and high-quality education to build health care professionals’ competencies in diabetes management and care, this is especially important for developing countries facing severe workforce shortages and increasing burden of chronic disease [5,39,40].

Digital education interventions in this review, although diverse in terms of the mode of delivery, mostly employed clinical scenarios for presentation of educational content. Furthermore, the included digital education interventions were mainly asynchronous and aimed at postregistration health care professionals. Although this digital education format may indeed be optimal for busy clinicians as part of their continuing professional development, there is scope for more research on other digital education formats as well as preregistration health care professionals [38,41,42].

### Limitations

There are limitations to the evidence included in this review. First, studies were too heterogeneous to be pooled. Second, many studies were at a high risk of biases such as selection and attrition bias. Third, satisfaction with digital education interventions may be overestimated by a heavy reliance on self-reported measures and a disproportionate focus on only the intervention group. Satisfaction is important in ensuring the success of digital education interventions as it impacts the user’s intention to sustain learning through digital means [33]. Therefore, alternative methods should be used to explore satisfaction with digital education interventions such as the actual time spent on digital learning or in-depth qualitative analyses on the perceptions of digital education. Finally, studies, in general, did not refer to a learning theory in the intervention design. Digital education presents a new model of learning where technological and Web-based learning expands and changes the paradigm of usual learning. Furthermore, the complexity of diabetes management may warrant a unique learning pedagogy. The use of technological or adult learning theories in the development of digital education interventions may improve the quality, reporting, and ingenuity of the digital education research if grounded in existing theoretical frameworks [34].

### Future Research

Digital education is rapidly transforming health professions training and is expected to gain even more prominence in the coming years. It is critical for digital education adoption and implementation to be guided by a robust evidence base. There is a need for more high-quality and standardized studies from a range of settings, including developing countries, which would focus on all aspects of diabetes management. Future research should also aim to assess the economic impact to inform planning, development, and adoption of digital health professions education interventions on diabetes management.

### Conclusions

Digital education holds the promise of a scalable and affordable approach to health professions education, with particular relevance to developing countries tackling severe shortage of skilled health care staff. In this review, we aimed to determine the effectiveness and cost-effectiveness of digital education for health professions education on diabetes management. We identified 12 studies showing that digital education is well-received and seems to improve knowledge and skills scores in health care professionals compared with traditional or usual education. Although digital education seems to be more effective, or not inferior to other forms of education on diabetes management, the paucity and low quality of data prevent us from making recommendations about its adoption. Future studies should focus on a range of outcomes using validated and standardized outcome measurements in different settings to improve the quality and credibility of evidence.

## Appendix

Multimedia Appendix 1 MEDLINE (Ovid) search strategy.

Multimedia Appendix 2 Forest plots for knowledge and skill outcomes.

Multimedia Appendix 3 Risk of bias assessments.

Multimedia Appendix 4 Detailed characteristics of the included studies.

## Abbreviations

PCP: primary care physician
RCT: randomized controlled trial
RR: risk ratio
SMD: standardized mean difference
