# Supplementary material for: Digital Health Professions Education on Diabetes Management: Systematic Review by the Digital Health Education Collaboration
Source: J Med Internet Res. 2019 Feb 21;21(2):e12997. doi: 10.2196/12997 (PMC6403527; doi:10.2196/12997)
Supplement: Multimedia Appendix 2 [file jmir_v21i2e12997_app2.pdf]

Multimedia Appendix 2: Forest plots for knowledge and skill outcomes

Study or Subgroup	Intervention Mean	Intervention SD	Intervention Total	Control Mean	Control SD	Control Total	Std. Mean Difference IV, Random, 95% CI
Chaikoolvatana 2007	57.1	4.2	43	47.9	4.4	40	2.12 [1.58, 2.66]
Desimone 2012	83	2.1	11	84	1.4	11	-0.54 [-1.39, 0.31]
Ngamruengphong 2015	65	11	20	27	16	19	2.72 [1.83, 3.62]
Sperl-Hillen 2014	5.3	1.8	92	4.1	1.6	128	0.71 [0.43, 0.99]

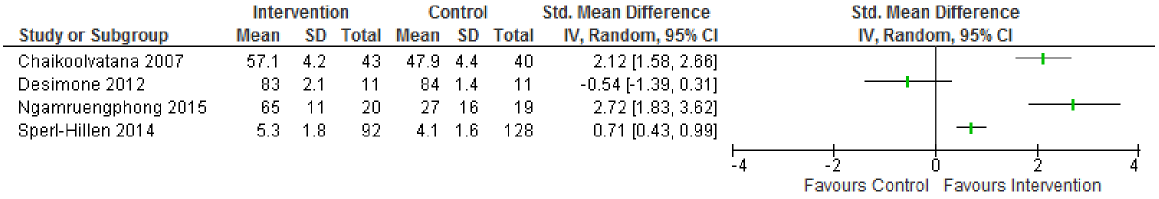

Figure A4.1: Forest plot comparing digital or blended education with traditional education, knowledge outcome (post-intervention data)

Study or Subgroup	Favours Intervention Events	Favours Intervention Total	Favours Control Events	Favours Control Total	Risk Ratio M-H, Random, 95% CI
**1.1.1 Shared agenda-setting**					
Gregory 2011a	15	29	2	29	7.50 [1.88, 29.89]
**1.1.2 Brainstroming**					
Gregory 2011b	7	29	1	29	7.00 [0.92, 53.36]
**1.1.3 Physical examination of lower limb**					
Hibbert 2013a	11	12	4	10	2.29 [1.05, 4.99]
**1.1.4 Diabetes history taking**					
Hibbert 2013b	10	12	2	10	4.17 [1.18, 14.77]
**1.1.5 Physical examination of thyroid disease**					
Hibbert 2013c	11	12	10	10	0.93 [0.73, 1.17]
**1.1.6 Scenario-1**					
Sperl-Hillen 2014a	38	177	3	164	11.74 [3.69, 37.29]
**1.1.7 Scenario-2**					
Sperl-Hillen 2014b	28	177	8	164	3.24 [1.52, 6.91]
**1.1.8 Scenario-3**					
Sperl-Hillen 2014c	85	177	17	164	4.63 [2.88, 7.45]
**1.1.9 Scenario-4**					
Sperl-Hillen 2014d	75	177	31	164	2.24 [1.56, 3.21]

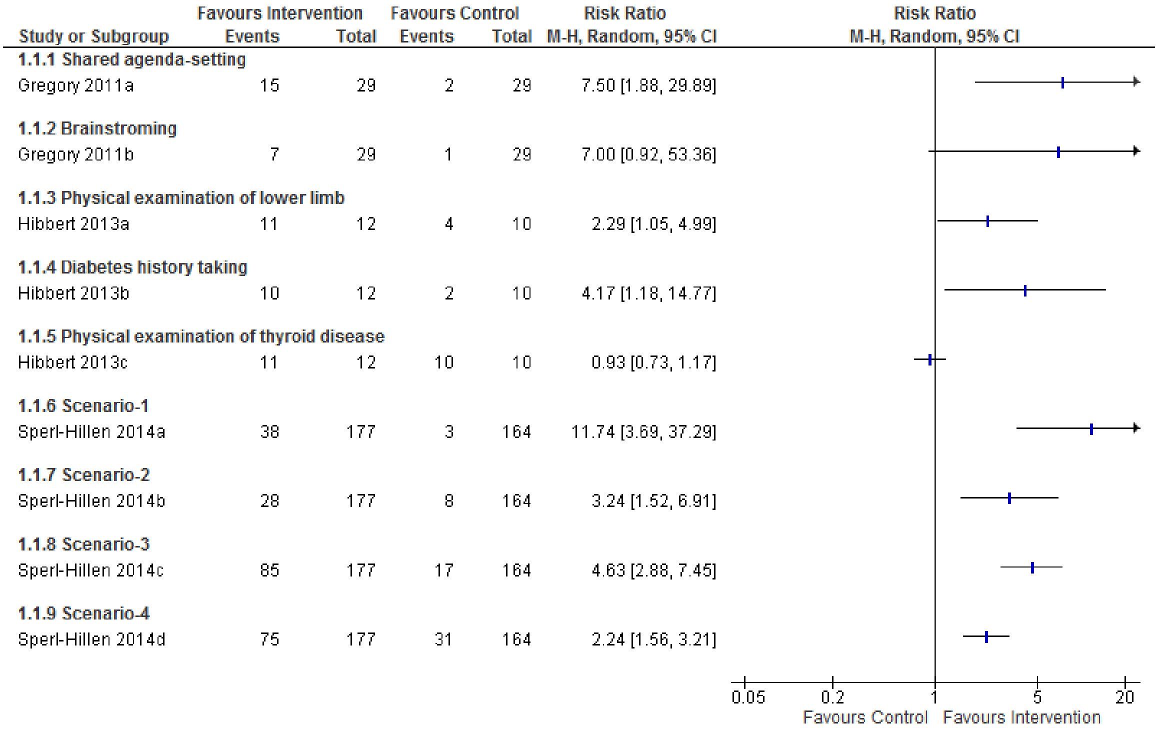

Figure A4.2: Forest plot comparing digital or blended education with traditional education, skills outcome (dichotomous, post-intervention data)

Study or Subgroup	Intervention Mean	Intervention SD	Intervention Total	Control Mean	Control SD	Control Total	Std. Mean Difference IV, Random, 95% CI
**1.1.1 Patient history writing skills**							
Chaikoolvatana 2007a	57.35	8.3	43	56.5	8.4	40	0.10 [-0.33, 0.53]
**1.1.2 SOAP note writing skills**							
Chaikoolvatana 2007b	70.65	8.4	43	64.03	8.5	40	0.78 [0.33, 1.22]
**1.1.3 Guiding style**							
Gregory 2011a	2.3	0.85	29	1.2	0.47	29	1.58 [0.99, 2.17]
**1.1.4 Evocation**							
Gregory 2011b	2	1.02	29	1.1	0.44	29	1.13 [0.57, 1.69]
**1.1.5 Collaboration**							
Gregory 2011c	2.3	0.84	29	1.5	0.63	29	1.06 [0.51, 1.62]
**1.1.6 Autonomy supportive**							
Gregory 2011d	2.4	0.82	29	1.3	0.6	29	1.51 [0.92, 2.10]
**1.1.7 Competence**							
Diehl 2015	40	15	94	34	15	76	0.40 [0.09, 0.70]

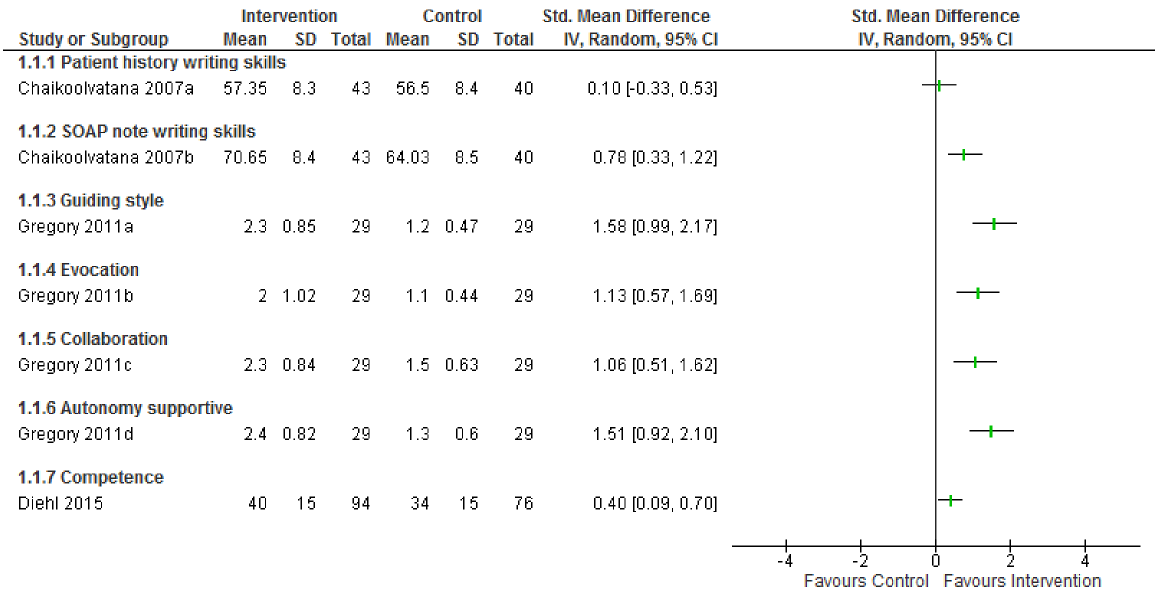

Figure A3.3: Forest plot comparing digital or blended education with traditional education, skills outcome (continuous, post-intervention data)

This is a Multimedia Appendix to a full manuscript published in the J Med Internet Res. For full copyright and citation information see http://dx.doi.org/10.2196/jmir.12997”
