# Supplementary material for: Digital Health Professions Education on Diabetes Management: Systematic Review by the Digital Health Education Collaboration
Source: J Med Internet Res. 2019 Feb 21;21(2):e12997. doi: 10.2196/12997 (PMC6403527; doi:10.2196/12997)
Supplement: Multimedia Appendix 3 [file jmir_v21i2e12997_app3.pdf]

Multimedia Appendix 3: Risk of bias assessments

**Study: Brendenkamp 2013**		
**Bias**	**Author's judgement**	**Support for judgement**
Random sequence generation (selection bias)	Unclear risk	Randomization was completed by staff educators who flipped a coin to assign the first participant to a learning strategy. However, randomization of the name list was not mentioned.
Allocation concealment (selection bias)	Unclear risk	Allocation concealment was not mentioned.
Blinding of participants and personnel (performance bias)	Low risk	Blinding of participants and personnel was not possible and it is unlikely that the lack of blinding would affect the results reported.
Blinding of outcome assessment (detection bias)	Unclear risk	No details given to enable judgement.
Incomplete outcome data (attrition bias)	Low risk	Attrition rate (2/96) is low. "The sample consisted of 96 nurses in a rural, mid-western acute care hospital." Table 2 and 3 showed 94 participants in the analysis.
Selective reporting (reporting bias)	Low risk	All pertinent outcomes mentioned in the methods section were reported.
Other bias	Unclear risk	"Nurses at the facility who did not participate in the study completed the education using simulation only. This might have encouraged some nurses to participate with the possibility of being randomized to the online strategy rather than simulation. While some nurses would have found the convenience of online learning to be a reason for choosing to participate, others might have avoided the study because of a dislike of online work."
**Study: Chaikoolvatana 2007**		
**Bias**	**Author's judgement**	**Support for judgement**
Random sequence generation (selection bias)	Unclear risk	Randomisation process was not mentioned
Allocation concealment (selection bias)	Unclear risk	Allocation concealment was not mentioned
Blinding of participants and personnel (performance bias)	Low risk	Blinding of participants and personnel was not possible and it is unlikely that the lack of blinding would affect the results reported
Blinding of outcome assessment (detection bias)	Unclear risk	No details given to enable judgement.
Incomplete outcome data (attrition bias)	Low risk	Pre and post-test numbers are the same. There is no attrition or drop-outs.
Selective reporting (reporting bias)	Low risk	All pertinent outcomes mentioned in the methods section were reported.
Other bias	High risk	Baseline differences were not measured. Small sample size and only patients who can speak English are interviewed, which may bias the generalizability of this study. Volunteer motivation may bias the implementation too.

Study: Desimone 2012		
**Bias**	**Author's judgement**	**Support for judgement**
Random sequence generation (selection bias)	Unclear risk	All 6 teams were randomised prior to the intervention. There is no more information regarding the process of randomisation.
Allocation concealment (selection bias)	Unclear risk	Allocation concealment was not mentioned
Blinding of participants and personnel (performance bias)	High risk	Participants cannot be blinded for digital education interventions, but the paper mentioned that cross-contamination was possible given the interaction of the residents.
Blinding of outcome assessment (detection bias)	Low risk	MCQ tests are less subjective. Also, blood glucose data were de-identified
Incomplete outcome data (attrition bias)	Low risk	There was no attrition for the knowledge test, given the small sample size. The reporting of attrition rate was unclear also.
Selective reporting (reporting bias)	Low risk	All pertinent outcomes mentioned in the methods section were reported.
Other bias	High risk	Possibility of cross contamination. The study lasted for one month and the same questionnaire was administered. Knowledge retention in a short period of time is high but may not be high in the long term. The I and C groups were also not tested for baseline differences.

Study: Diehl 2015		
**Bias**	**Author's judgement**	**Support for judgement**
Random sequence generation (selection bias)	Low risk	A simple randomization process was conducted using a random number generator.
Allocation concealment (selection bias)	Low risk	Allocation number was done using random online number generator - www.random.org - the moment when a participant signed the Informed Consent Form
Blinding of participants and personnel (performance bias)	Low risk	Blinding of participants and personnel was not possible and it is unlikely that the lack of blinding would affect the results reported. Digital education interventions cannot be blinded
Blinding of outcome assessment (detection bias)	Unclear risk	Outcomes were assessed using MCQs, so it is unlikely for detection bias to occur.
Incomplete outcome data (attrition bias)	Unclear risk	Attrition was moderate "In game group, 69 of 88 (78%) completed the intervention, compared to 65 of 73 (89%) in control group, with no difference in applicability."
Selective reporting (reporting bias)	Low risk	This study on Educational Efficacy Assessment of a Serious Game to Teach Insulin Therapy to Primary Care Physicians was a randomised controlled trial (Clinicaltrials.gov: NCT01759953). All pertinent outcomes mentioned in the methods section of the paper were reported.
Other bias	Low risk	

Study: Estrada 2011		
**Bias**	**Author's judgement**	**Support for judgement**
Random sequence generation (selection bias)	Low risk	Block randomisation was carried out. Unit of randomisation was physician and only one physician per practice could enrol in the study. Random sequence was not mentioned.
Allocation concealment (selection bias)	Low risk	Randomisation was concealed to the investigators and statisticians.
Blinding of participants and personnel (performance bias)	Low risk	Blinding of participants and personnel was not possible and it is unlikely that the lack of blinding would affect the results reported.
Blinding of outcome assessment (detection bias)	Low risk	Data abstraction was performed by trained personnel on blinded records sent to the study center (or abstracted on site) (pg 683, data sources).
Incomplete outcome data (attrition bias)	High risk	Attrition rate is high as >50% of the randomised practices were not analysed
Selective reporting (reporting bias)	High risk	The Rural Diabetes Online Care (R-DOC) study was a cluster-randomised trial (Clinical Trials.gov identifier: NCT00403091). Primary outcomes such as A1c, blood Pressure, lipids mentioned in the study record are reported in the study. However some secondary outcomes listed in the study report such as eye screening, foot exam, kidney disease monitored, dietary or exercise advice, smoking cessation advice are not reported in the study.
Other bias	High risk	Table 1 shows the baseline characteristics of patients, physician characteristics not reported. The non-random sampling of physicians may have introduced selection bias. Second, physicians were asked to provide records of consecutively seen patients, and while we were unable to monitor compliance with this request, the wide range of A1c, BP and LDL values suggests that not only well-controlled patients were selected, but poorly-controlled patients as well. About 10 or 15 records did not represent the physician's diabetes patients in general. The high attrition may have introduced biases.
**Study: Gregory 2011**		
**Bias**	**Author's judgement**	**Support for judgement**
Random sequence generation (selection bias)	Low risk	There is for the design of the randomisation protocol. Half of the trial centres are randomised to the intervention arm, and half to the control arm. Teams were recruited and randomisation was optimally balanced for population size, separate/transition clinic and presence or absence of any paediatric-trained nurse within the clinic.
Allocation concealment (selection bias)	High risk	In order to reduce allocation knowledge bias, unblinded cluster randomised trials should aim to recruit all subjects before the allocation of the centres is revealed. In practice this was not always possible. In DEPICTED, 213/693 (30.7%) of subjects on centre eligibility lists were approached and consented prior to revealing allocation to the centres.
Blinding of participants and personnel (performance bias)	Low risk	Blinding of participants and personnel was not possible and it is unlikely that the lack of blinding would affect the results reported.

Blinding of outcome assessment (detection bias)	Unclear risk	Recordings from providers were assessed for mean guiding scores but blinding of assessment was not mentioned, although there were two independent assessors to reduce subjectivity in interpretation.
Incomplete outcome data (attrition bias)	Low risk	There is 95.3% follow-up while the study design is planned for 22% attrition.
Selective reporting (reporting bias)	Low risk	The outcomes were clearly reported as stated by the objectives
Other bias	Unclear risk	
**Study: Hibbert 2013**		
**Bias**	**Author's judgement**	**Support for judgement**
Random sequence generation (selection bias)	Unclear risk	Participants were randomised to gain access to either intervention or usual revision, but the randomisation process was not reported.
Allocation concealment (selection bias)	Unclear risk	Allocation concealment is not mentioned.
Blinding of participants and personnel (performance bias)	Low risk	Digital education cannot be blinded.
Blinding of outcome assessment (detection bias)	Low risk	Assessors were blinded to student randomization status.
Incomplete outcome data (attrition bias)	Low risk	There is no attrition as only 22 students are randomised.
Selective reporting (reporting bias)	Low risk	The authors measured LLE, DMH and TE as stated in the objectives
Other bias	Unclear risk	Baseline was not adjusted in the analysis
**Study: Ngamruengphong 2015**		
**Bias**	**Author's judgement**	**Support for judgement**
Random sequence generation (selection bias)	Unclear risk	The PC residents at one teaching hospital were then randomly assigned in a 1:1 ratio to one of two groups: a control group (C) and an educational intervention (E) group. (pg 511, phase 2, methods). Random sequence is not mentioned.
Allocation concealment (selection bias)	Unclear risk	Allocation concealment was not mentioned
Blinding of participants and personnel (performance bias)	Low risk	Blinding of participants and personnel was not possible and it is unlikely that the lack of blinding would affect the results reported.
Blinding of outcome assessment (detection bias)	Low risk	No details given to enable judgement. Assessment is conducted via an online survey, so outcome assessment cannot be biased.
Incomplete outcome data (attrition bias)	Low risk	100% completed, no attrition or drop out.
Selective reporting (reporting bias)	High risk	Control data was not reported for post-test comparison (fig 2, pg 513)
Other bias	High risk	The intervention group received the survey immediately after the intervention and both groups were given the survey again in 6 months. The intervention group may have done better because they did the survey one more time compared to the control group. Since the study relied on self-reported survey, there may be recall bias.

Study: O'connor 2009		
**Bias**	**Author's judgement**	**Support for judgement**
Random sequence generation (selection bias)	High risk	Only 67/122 of identified physicians consented to the study. This may have biased the study with more cooperative participating physicians.
Allocation concealment (selection bias)	Unclear risk	Allocation concealment was not mentioned. Physicians were blocked into groups of three base on their specialty and the number of patients under their care.
Blinding of participants and personnel (performance bias)	Low risk	Blinding of participants and personnel was not possible and it is unlikely that the lack of blinding would affect the results reported.
Blinding of outcome assessment (detection bias)	Low risk	Outcomes were obtained from patient's records (measured during routine check-up), which cannot be manipulated by researchers. Hence, bias in assessment is low.
Incomplete outcome data (attrition bias)	Low risk	Although attrition rate is slightly high among physicians (8/65), it occurred evenly among groups and the final number still met the minimum sample size for power detection. Such attrition may affect the generalizability of the results rather than the reliability of the results.
Selective reporting (reporting bias)	Low risk	All pertinent outcomes mentioned in the methods section were reported.
Other bias	Unclear risk	Analysis should be intention to treat (IIT) instead of per protocol in a trial but IIT was not used here.
**Study: Schroter 2011**		
**Bias**	**Author's judgement**	**Support for judgement**
Random sequence generation (selection bias)	Low risk	Randomisation was carried out by an independent statistician. This is a covariate adjusted block randomisation.
Allocation concealment (selection bias)	Low risk	One thousand allocations with the greatest degree of balance were identified and passed to an independent statistician within the South East Wales Trials Unit (SEWTU) at Cardiff University, who randomly selected a single allocation for each block. This was then returned to the trial statistician (RP) and the study database manager informed of the allocations.
Blinding of participants and personnel (performance bias)	Low risk	Blinding of participants and personnel was not possible and it is unlikely that the lack of blinding would affect the results reported.
Blinding of outcome assessment (detection bias)	Low risk	The analysis was conducted by the trial statistician blinded to group allocation of participants.
Incomplete outcome data (attrition bias)	Low risk	All analyses followed the intention to treat (ITT) principle and groups were analysed as randomised. Missing Test 2 scores were assumed to have remained unchanged for the ITT analysis. A complete case analysis (CCA) was also carried out excluding those missing follow-up test scores. Attrition rates were similar in both groups (pg 6, discussion). Authors have also included additional unplanned analysis (pg 4).
Selective reporting (reporting bias)	Low risk	All pertinent outcomes mentioned in the methods section were reported.
Other bias	Low risk	

Study: Sperl-Hillen 2010		
**Bias**	**Author's judgement**	**Support for judgement**
Random sequence generation (selection bias)	Unclear risk	PCPs were block randomized on the basis of baseline quality of diabetes care and number of consenting primary care physicians (PCPs). Quality is not defined while consentment of PCPs may be biased to other external factors.
Allocation concealment (selection bias)	Unclear risk	Allocation concealment is not mentioned.
Blinding of participants and personnel (performance bias)	Low risk	Blinding of participants and personnel was not possible, and it is unlikely that the lack of blinding would affect the results reported.
Blinding of outcome assessment (detection bias)	Unclear risk	HbA1c level measurements have low risks as biomarkers. Ways of measuring the satisfaction survey was not explicitly mentioned.
Incomplete outcome data (attrition bias)	Low risk	There were 0 lost to follow-up in the intervention and 1 PCP transferred to other administrative duties in the control group.
Selective reporting (reporting bias)	Low risk	All pertinent outcomes mentioned in the methods section were reported.
Other bias	Unclear risk	There may be some selection bias while selecting initial PCPs to be randomised (11 PCPs were randomised but how it was randomised was not mentioned). There may also self-reported bias for the satisfaction survey as it is unsure how the survey is administered.
**Study: Sperl-Hillen 2014**		
**Bias**	**Author's judgement**	**Support for judgement**
Random sequence generation (selection bias)	Low risk	Ten residency programs with 177 consented residents were subsequently assigned using a random number generator to SimDE and 9 programs with 164 consented residents to the control group (CG)
Allocation concealment (selection bias)	Unclear risk	The identity of participating residents was kept confidential from residency program staff, but the residency program staff's role is unclear.
Blinding of participants and personnel (performance bias)	Low risk	Blinding of participants and personnel was not possible and it is unlikely that the lack of blinding would affect the results reported.
Blinding of outcome assessment (detection bias)	Unclear risk	The test was developed by the study team and tested with practicing providers and certified diabetes nurse specialists to ensure clarity but it is unsure whether the assessors were blinded to the allocation.
Incomplete outcome data (attrition bias)	High risk	Attrition is imbalanced across intervention and control group. The intervention group had almost 50% attrition (high) while the control group had about 16.4% attrition.
Selective reporting (reporting bias)	Low risk	All pertinent outcomes mentioned in the methods section were reported.
Other bias	Unclear risk	There may be selection bias as about half the participants did not consent/volunteer to the study at the beginning.

Study ID	Recruitment bias	Baseline imbalance	Loss of clusters	Incorrect analysis	Comparability with individual trials
Sperl-Hillen 2010	Low	High	Low	Low	Unclear
	Eleven HPMG clinics were randomly selected and block randomized on the basis of baseline quality of diabetes care and number of consenting primary care physicians (PCPs) to either receive or not receive the intervention.	Randomization at the clinic level resulted in an intervention arm with a higher proportion of younger and male patients.	There was no loss of clusters. (Figure 1, pg. 1728)	The study considered intraclass correlation (ICC) in the analysis	Comparison between cRCT and RCT could not be made due to heterogeneity in the intervention content.
Sperl-Hillen 2014	Low	High	Low	Low	Unclear
	Invitation to the residency programme was conducted nationally through nationally by e-mail, phone, and listserv postings.	Baseline differences were observed for Ethnicity/ Race and knowledge using drugs to manage diabetes and knowledge interpreting SMBG	There was no loss of clusters. (Figure 1, pg. 1665)	Group comparisons were analyzed using generalized linear mixed models, controlling for clustering of residents within residency programs and differences in baseline knowledge.	Comparison between cRCT and RCT could not be made due to heterogeneity in the intervention content.
Gregory 2011	Low	Low	Low	Low	Unclear
	Allocation was based on clusters (i.e. paediatric diabetes services), with half randomized to the intervention and half to the control arm, in three phases, balanced for patient list size.	Baseline data indicate that the randomization achieved adequate balance for all demographic variables including the primary outcome, HbA1c levels. There were slightly more males in the intervention	There was no loss of clusters. (Figure 7, pg. 72)	Cluster level Cluster-level balance was examined for patient demographic data. Standard deviations were adjusted to account for clustering of responses within services through inflation by the intracluster	Comparison between cRCT and RCT could not be made due to heterogeneity in the intervention content.

		arm but adjusting for gender in the primary analysis did not influence the result.		correlation coefficient (ICC).	

This is a Multimedia Appendix to a full manuscript published in the J Med Internet Res. For full copyright and citation information see http://dx.doi.org/10.2196/jmir.12997”
