# Supplementary material for: Digital Health Professions Education on Diabetes Management: Systematic Review by the Digital Health Education Collaboration
Source: J Med Internet Res. 2019 Feb 21;21(2):e12997. doi: 10.2196/12997 (PMC6403527; doi:10.2196/12997)
Supplement: Multimedia Appendix 4 [file jmir_v21i2e12997_app4.pdf]

Multimedia Appendix 4. Detailed characteristics of the included studies

Study; design; Country; Income	Learning modality	Type and number of participants	Intervention duration	Knowledge	Skill	Patient clinical outcomes	Economic impact
Digital education vs. Traditional education							
Chaikoolvatana 2007 [1]; RCT; Thailand; MIC	I: Computer based learning (CBL)<br><br>C: Face-to-face lectures	Final year pharmacy students<br>I: 43, C: 40	I: 2 hours; C: 2 3-hour sessions (Over 2 months)	Post-intervention MCQ on diabetes management<br><br>SMD =2.14[a], 95% CI [1.60, 2.68]	Patient history taking skills post-intervention<br>SMD = 0.10<br>95% CI [-0.33, 0.53]<br>SOAP note writing skills post-intervention<br>SMD = 0.78[a]<br>95% CI [0.33, 1.22]		
Desimone 2012 [2]; RCT; United States; HIC	I: PDA version education materials<br><br>C: Printed materials	Internal medicine residents<br>I: 11, C: 11	(Over 1 month)	Diabetes knowledge post-intervention test<br>SMD = -0.56, 95% CI [-1.41, 0.29]			
Diehl 2017 [3]; RCT; Brazil; MIC	I: Online game<br><br>C: Face-to-face lectures and activities	Primary Care Physicians (PCP)<br>I: 94, C: 76	4 hours<br>(Over 3 months)		Absolute increase in insulin prescription competence post intervention<br>SMD = 0.4[a]<br>95% CI [0.09, 0.71]		

Hibbert 2013 [4]; RCT; Australia; HIC	I: Training Video<br><br>C: No intervention (usual revision)	2nd year medical students<br>I: 12, C: 10	Over 2 Weeks		Proportion of competent students:<br>Lower Limb Complication Exam (LLE):<br>RR = 2.29[a]<br>95% CI [1.05, 4.99];<br>History taking (DMH):<br>RR= 4.17[a]<br>95% CI [1.18, 14.8];<br>Thyroid exam (TE):<br>RR = 0.93<br>95% CI [0.73, 1.17]		
Sperl-Hillen 2010 [5]; cRCT; United States; HIC	I: Simulation Software<br><br>C: No intervention	PCPs, Patients<br>I: (20, 1847), C: (21,1570)	5.5 days<br>(Over 6 months)			Proportion of patients with optimal control:<br>HbA1c (<7%):<br>RR = 1.30[a]<br>95% CI [1.16, 1.46];<br>SPB (<130 mmHg):<br>RR = 0.95<br>95% CI [0.89, 1.01];<br>DPB (<80 mmHg):<br>RR = 1.03<br>95% CI [0.97, 1.09];<br>LDL (<100 mg/dl):<br>RR = 1.00<br>95% CI [0.92, 1.08]	Total costs ($(SE)) including intervention and health care costs were estimated to be US$71 (142) lower per patient in the intervention clinic. The difference was not significant.

Sperl-Hillen 2014 [6]; cRCT; United States; HIC	I: Simulation software<br><br>C: No intervention (Not assigned learning cases)	Family/ internal medicine residents<br>I: (10, 177), C: (9, 164)	Over 6 months	Case-based post-intervention knowledge test SMD = 0.7[a]<br>95% CI [0.48, 0.92]	Proportion of patients with optimal control in 4 hypothetical clinical cases:<br>Case 1: RR = 11.8[a]<br>95% CI [3.69, 37.3]<br>Case 2: RR = 3.2<br>95% CI [1.52, 6.91]<br>Case 3: RR = 4.6[a])<br>95% CI [2.88, 7.45]<br>Case 4: RR = 2.2[a]<br>95% CI [1.56, 3.21]		
Blended learning vs. Traditional education							
Gregory 2011 [7]; cRCT; United Kingdom; HIC	I: Web-based intervention and practical workshops<br><br>C: No intervention	Paediatric doctors, nurses, psychologists, dieticians<br>I: (13 sites; 356 patients); C: (13 sites; 333 patients)	Over 12 months		Global rating of behavioural change guiding style SMD = 1.58[a]<br>95% CI [0.99, 2.17]<br><br>Proportion of HCPs with Shared agenda setting RR = 7.49[a]<br>95% CI [1.88, 29.9]	Mean difference in (HbA1c)<br>MD = 0.01[a]<br>95% CI [-0.02, 0.04]	Mean Total NHS cost (including training) (I: £1393.38 (£1298.69); C: £1209.42 (£676.41)
Ngamruengphong 2011 [8]; RCT; United States; HIC	I: Standard education + 30-minute didactic lecture, a pocket card, and monthly e-mail reminders that consisted of the lecture content<br><br>C: Standard residency education	Primary care residents<br>I: 20, C: 19	Over 2 months	Post-intervention knowledge test SMD = 1.98[a]<br>95% CI [1.21, 2.74]			

Digital education vs. Digital education							
Billue 2012 [9]; RCT United States; HIC<br><br>Estrada 2011[10]; RCT United States; HIC<br><br>Crenshaw 2010[11]; RCT; United States; HIC	I: Web-based intervention with feedback<br><br>C: Web-based intervention without feedback	Family/ general/ internal med physicians<br>I: 48, C: 47	Over 2 years			Proportion of patients with optimal control of:<br>HbA1C (<7%):<br>RR = 1.10<br>95% CI [0.98, 1.24];<br>BP (<130/80mmHg):<br>RR = 1.10<br>95% CI [0.92, 1.32];<br>LDL (<100mg/dl):<br>RR = 0.95<br>95% CI [0.86, 1.05]<br><br>Rate of medication intensification for:<br>HbA1C control (<7%):<br>RR = 1.05<br>95% CI [0.88, 1.24];<br>BP control (<130/80mmHg:<br>RR = 0.98<br>95% CI [0.75, 1.27];<br>LDL control (<100mg/dl):<br>RR = 1.35<br>95% CI [0.94, 1.93]	

Brendenkamp 2013 [12]; RCT; United States; HIC	I: Simulation (High fidelity Mannequin)<br><br>C: Web-based intervention	Staff nurses<br><br>I: 47, C: 49	Not reported	Post-intervention hypoglycaemia – knowledge test SMD = -0.37 95% CI [-0.77, 0.033]			
Schroter 2011 [13]; RCT; United Kingdom; HIC	I: Web-based learning + Diabetes Needs assessment tool (DNAT)<br><br>C: Web-based learning without DNAT	Diabetes doctors and nurses I: 499, C: 498	Over 4 months	Post-intervention MCQs Diabetes Knowledge SMD = -0.06 95% CI [-0.19, 0.05]			
Blended learning vs. Digital education vs. Traditional education							

O'Connor 2009 [14]; RCT; United States; HIC	Group A: No intervention<br>Group B: Simulated web-based learning<br>Group C: simulated case-based physician learning + physician opinion leader feedback	Group (No. of Primary care physicians, No. of patients)<br>A: (100, 691), B: (100, 725), C: (99, 604)	NR			Mean (pre-post) change in HbA1c control[a]:<br>A = 0.06, B = -0.01, C = 0.18;<br>LDL (mg/dl) control:<br>A = -4.1, B = -0.2.4, C = -3.7<br><br>Rate of medication intensification:<br>For HbA1c control (%):<br>$RR_{(B/A)}$ =1.03; 95% CI [0.69, 1.55]<br>$RR_{(C/A)}$ =1.16; 95% CI [0.80, 1.74]<br>For LDL (mg/dl) control:<br>$RR_{(B/A)}$ = 1.16; 95% CI [0.96, 1.41]<br>$RR_{(C/A)}$ =1.05; 95% CI [0.85, 1.29]	

*p<0.05, (Taken as statistically significant with better outcomes for the intervention group)
[#]Competence was computed as a combination of Knowledge and Skills, assessed as figurative knowledge (9 MCQS) about insulin and clinical case vignettes (11 MCQs) in Diehl 2015's study.

Abbreviations: Confidence interval (CI); cluster RCT (cRCT); Control Group (C); Diastolic blood pressure (DPB); High income country (HIC); Intention to treat (ITT); Intervention group (I); Low-density lipoprotein (LDL); Middle Income country (MIC); Not reported (NR); Personal Digital Assistance (PDA); Primary care physicians (PCPs); Randomised controlled trial (RCT); Risk ratio (RR); Standard deviation (SD); Standardised mean difference (SMD); SimDE Simulated Diabetes Education; Subjective, Objective, Assessment; Plan (SOAP); Systolic blood pressure (SBP)
Differences of continuous outcomes were presented in Standardized mean differences (SMD) while dichotomous outcomes with proportions were presented in risk ratios (RR)

This is a Multimedia Appendix to a full manuscript published in the J Med Internet Res. For full copyright and citation information see http://dx.doi.org/10.2196/jmir.12997"

References

1. *Chaikoolvatana A, Haddawyvaluation OTEOACL(PIDM. J Med Assoc Thai. Journal of the Medical Association of Thailand 2007 Jul; 90(7):1430-4*
2. *Desimone ME, Blank GE, Virji M, Donihi A, DiNardo M, Simak DM, Buranosky R, Korytkowski MT. Effect of an educational Inpatient Diabetes Management Program on medical resident knowledge and measures of glycemic control: a randomized controlled trial. Endocr Pract 2012 Mar; 18(2):238-49*
3. *Diehl LA, Souza RM, Gordan PA, Esteves RZ, Coelho ICM. InsuOnline, an Electronic Game for Medical Education on Insulin Therapy: A Randomized Controlled Trial With Primary Care Physicians. J Med Internet Res 2017 Dec 09; 19(3):e72*
4. *Hibbert E, Lambert T, Carter J, Learoyd D, Twigg S, Clarke S. A randomized controlled pilot trial comparing the impact of access to clinical endocrinology video demonstrations with access to usual revision resources on medical student performance of clinical endocrinology skills. BMC Med Educ 2013 Oct 03; 13:135*
5. *Sperl-Hillen JM, O'Connor PJ, Rush WA, Johnson PE, Gilmer T, Biltz G, Asche SE, Ekstrom HL. Simulated physician learning program improves glucose control in adults with diabetes. Diabetes Care 2010 Aug; 33(8):1727-33*
6. *Sperl-Hillen J, O'Connor PJ, Ekstrom HL, Rush WA, Asche SE, Fernandes OD, Appana D, Amundson GH, Johnson PE, Curran DM. Educating resident physicians using virtual case-based simulation improves diabetes management: a randomized controlled trial. Acad Med 2014 Dec; 89(12):1664-73*
7. *Gregory J, Robling M, Bennert K, Channon S, Cohen D, Crowne E. Development and evaluation by a cluster randomised trial of a psychosocial intervention in children and teenagers experiencing diabetes: the DEPICTED study. Health Technol Assess 2011 Aug; 15(29):1-202*
8. *Ngamruengphong S, Horsley-Silva J, Hines S, Pungpapong S, Patel T, Keaveny A. Educational Intervention in Primary Care Residents' KnowledgePerformance of Hepatitis B Vaccination in Patients with Diabetes Mellitus. South Med J 2015 Sept 09; 108(9):510-5*
9. *Billue K, Safford M, Salanitro A, Houston T, Curry W, Kim Y. Medication intensification in diabetes in rural primary care: a cluster-randomised effectiveness trial. BMJ open 2012 Sept; 2(5)*
10. *Estrada CA, Safford MM, Salanitro AH, Houston TK, Curry W, Williams JH, Ovalle F, Kim Y, Foster P, Allison JJ. A web-based diabetes intervention for physician: a cluster-randomized effectiveness trial. Int J Qual Health Care 2011 Dec; 23(6):682-9*
11. *Crenshaw K, Curry W, Salanitro A, Safford M, Houston T, Allison J. Is physician engagement with Web-based CME associated with patients' baseline hemoglobin A1c levels? The Rural Diabetes Online Care study. Acad Med 2010 Sept; 85(9):1511-7*
12. *Bredenkamp N. Simulation versus online learning: effects on knowledge acquisition, knowledge retention, and perceived effectiveness. In: University of Northern Colorado PhD Dissertation. Denver: University of Northern Colorado; 2013 May.*
13. *Schroter S, Jenkins RD, Playle RA, Walsh KM, Probert C, Kellner T, Arnhofer G, Owens DR. Evaluation of an online interactive Diabetes Needs Assessment Tool (DNAT) versus online self-directed learning: a randomised controlled trial. BMC Med Educ 2011 Jun 16; 11:35*
14. *O'Connor P, Sperl-Hillen J, Johnson P, Rush W, Asche S, Dutta P, Biltz George R. Simulated physician learning intervention to improve safety and quality of diabetes care: a randomized trial. Diabetes Care 2009 Apr; 32(4):585-90*
